# N-glycosylation at the receptor binding site drives differences in receptor binding specificity between influenza B virus lineages

**DOI:** 10.1128/jvi.01039-25

**Published:** 2025-11-05

**Authors:** Caroline K. Page, M. H. M. Mubassir, Pradeep Chopra, Lindsey Claire Gay, Ginger Geiger, Sean D. Ray, Justin D. Shepard, Rose J. Miller, Daniel Perez, Justin Bahl, Gerardus Josephus Boons, Stephen Mark Tompkins

**Affiliations:** 1 Center for Vaccines and Immunology, University of Georgia 822673, Athens, Georgia, USA; 2 Center for Influenza Disease and Emergence Response (CIDER), University of Georgia 1355, Athens, Georgia, USA; 3 Department of Infectious Diseases, University of Georgia 551782, Athens, Georgia, USA; 4 Institute of Bioinformatics, University of Georgia 1355, Athens, Georgia, USA; 5 Complex Carbohydrate Research Center, University of Georgia 123423, Athens, Georgia, USA; 6 Department of Population Health, University of Georgia 308501, Athens, Georgia, USA; 7 College of Public Health, University of Georgia 189617, Athens, Georgia, USA; 8 Chemical Biology & Drug Discovery, Utrecht Institute for Pharmaceutical Sciences, Utrecht University 8125, Utrecht, the Netherlands; 9 Department of Chemistry, University of Georgia 735294, Athens, Georgia, USA; Cornell University Baker Institute for Animal Health, Ithaca, New York, USA

**Keywords:** Yamagata, Victoria, glycosylation, FLUBV, receptor specificity, influenza

## Abstract

Receptor specificity plays a critical role in influenza virus host tropism and pathogenesis. Influenza A and influenza B viruses (FLUAVs and FLUBVs, respectively) utilize N-glycans with terminal sialic acids on glycoproteins decorating the surface of mucosal epithelial cells as receptors for the virus hemagglutinin (HA) protein. For FLUAVs, the specificity of HA binding to distinct sialic acid linkages on host glycans is a major determinant of species specificity. Amino acid motifs and N-linked glycosylation sites influencing FLUAV HA receptor specificity are well defined. In contrast, considerably less is known regarding determinants of FLUBV receptor specificity, despite its significant contribution to the global influenza disease burden and unique restriction for human hosts. To address this knowledge gap, we utilize microarrays populated with glycans resembling structures found in the respiratory tract to comprehensively define the receptor binding profiles of FLUBVs from different decades and lineages and confirm these results with complementary virus–glycan binding assays. Using wild-type and reverse genetics FLUBVs having singular mutations in the HA receptor binding site (RBS), as well as structural models, we identify an N-glycosylation site at amino acid 196 within the RBS that determines the breadth of HA binding to terminal sialic acids. The definition of the presence of an N-linked glycan as the primary determinant for FLUBV receptor specificity provides a clear mechanism for lineage-specific differences in HA receptor binding. This may help explain the distinct tropism observed between Victoria and Yamagata lineage FLUBVs and provide insights into the disappearance of the Yamagata lineage.

**IMPORTANCE:**

Influenza B viruses (FLUBVs) are a major cause of human respiratory disease, but the molecular determinants influencing receptor specificity for the hemagglutinin protein remain largely undefined. We defined the receptor specificity of a panel of Early, Victoria, and Yamagata lineage viruses spanning over 50 years and showed that Victoria lineage viruses can have expanded receptor specificity, compared to Yamagata lineage viruses. We identified a critical N-glycosylation site within the hemagglutinin that regulates hemagglutinin binding to sialic acid receptors on host cells. Recent successful subclades of the Victoria lineage viruses lost this glycosylation site, enabling binding to both human-type and avian-type sialic acid receptors, which may influence respiratory tract tropism. These viruses also showed increased endemic activity. The expanded receptor tropism of Victoria lineage viruses may have fitness benefits, helping to explain epidemiologic features of the lineage, perhaps contributing to the lineage’s recent success, while the Yamagata lineage appears to have become extinct.

## INTRODUCTION

Influenza B viruses (FLUBVs) have circulated exclusively in humans for over 80 years, contributing substantially to the influenza disease burden, particularly in children and the elderly (1–4). FLUBVs are classified into two antigenically distinct lineages, Victoria and Yamagata, which diverged in the 1970s (5–9). While the evolutionary dynamics of FLUBVs are complex, their separation is ultimately defined by differences in their hemagglutinin (HA) protein (7). Unlike influenza A viruses (FLUAVs), which infect a broad range of avian and mammalian species and pose a pandemic risk through zoonotic transmission, FLUBVs circulate exclusively in humans, causing only epidemics (10–12). While they lack the antigenic and host diversity of FLUAVs, FLUBVs nonetheless undergo antigenic variation through reassortment with co-circulating viruses and the accumulation of mutations, which together drive antigenic drift (13, 14). These processes contribute to the divergence of the lineages and have been associated with increased epidemic activity (14). Notably, despite co-circulating for nearly four decades, Yamagata viruses have not been detected since March 2020, ultimately leading the World Health Organization to recommend their removal from seasonal influenza vaccine formulations (15, 16).

A key determinant of FLUAV and FLUBV receptor specificity is the ability of the HA protein to bind *N*-acetylneuraminic acid (Neu5Ac) in the receptor binding pocket. Glycoproteins on the cells lining the upper and lower respiratory tracts of host species have poly-N-acetyllactosamine structures terminating with either α2,6 or α2,3 linked sialic acids (Sia), which FLUAVs and FLUBVs variably utilize for attachment and infection (12). The receptor specificity of FLUAVs has been extensively studied to help define factors contributing to host range and the risk of viruses crossing species barriers. In contrast, relatively few studies have defined the receptor specificity and tropism of FLUBVs (17). Wang et al. analyzed glycan-binding properties of FLUBVs using clinical isolates from Taiwan collected between 2001 and 2007. They found that Yamagata viruses exclusively bound to α2,6-linked Neu5Ac, while some Victoria viruses bound both α2,6 and α2,3-linked Neu5Ac, and others bound only α2,6-linked Neu5Ac (18). A correlation between the expanded glycan binding profile of Victoria lineage viruses and year of isolation suggested viral evolution might influence receptor specificity. Despite these observations, most studies defining FLUBV receptor binding focused on earlier viral isolates, often from limited geographic regions, leaving the receptor binding characteristics of recent, globally diverse FLUBV strains largely undefined.

The FLUBV HA protein contains a receptor-binding site (RBS) that includes parts of the 190-helix, 240-loop, and 140-loop (19). Four key residues, namely Phe-95, Trp-158, His-191, and Tyr-202, form the base of the RBS and are conserved across all FLUBVs (20, 21). Structural comparisons between the HA of B/Hong Kong/8/73 and influenza A H3 HA (X31) highlighted conserved residues, including Thr-139, Ser-140, and Gly-141, within the 190-helix, and Pro-238 and Ser-240 within the 240-loop, which interact with Neu5Ac (7). Single amino acid mutations within the globular head of the HA can alter receptor specificity and the avidity of the HA-Sia interaction, particularly if the mutation affects a glycosylation site defined as N-X-S/T (22, 23). Glycosylation of influenza HA plays an important role in the virus’s evolution, enabling immune evasion by shielding antigenic sites, altering receptor binding, and affecting host specificity (24–27). Experimentally passaging FLUBVs in embryonated chicken eggs induces mutations at residues 194–196, which alter an N-glycosylation site and increase the virus’s affinity for α2,3-linked Neu5Ac (28–32). While this augmentation is artificial, natural differences in glycosylation between the lineages exist, and it remains unknown whether mutations separating the lineages contribute to their differences in sialic acid receptor binding.

Extensive research has characterized how amino acid mutations within the HA, changes in glycosylation sites, and other molecular variations drive shifts in receptor specificity for FLUAVs; however, significant gaps remain in our understanding of these factors for FLUBV receptor specificity (23, 33). While structural and predictive models provide valuable insights into the molecular factors affecting FLUBV receptor specificity, experimental data to substantiate these models are limited (19, 34, 35). To address this knowledge gap, we utilized microarrays populated with glycans that mimic structures found in respiratory tissue to comprehensively define the receptor binding profiles of FLUBVs from different decades, lineages, and clades. Importantly, all the FLUBVs tested were isolated and cultured exclusively in mammalian cell substrates, avoiding the potential for egg-adaptive mutations influencing the results. Using reverse genetics viruses with single-point mutations and molecular dynamics, we examined the role of the 196 N-glycosylation site as a molecular determinant of FLUBV receptor specificity. Our findings show that this site is critical in determining the breadth of Victoria and Yamagata lineage binding to terminal sialic acid conformations. This data enhances our understanding of FLUBV glycan–host interactions and provides a clear mechanism for lineage-specific differences in HA receptor binding.

## RESULTS

### Genetic variation affecting the receptor-binding domain of influenza B viruses

**Fig 1:**
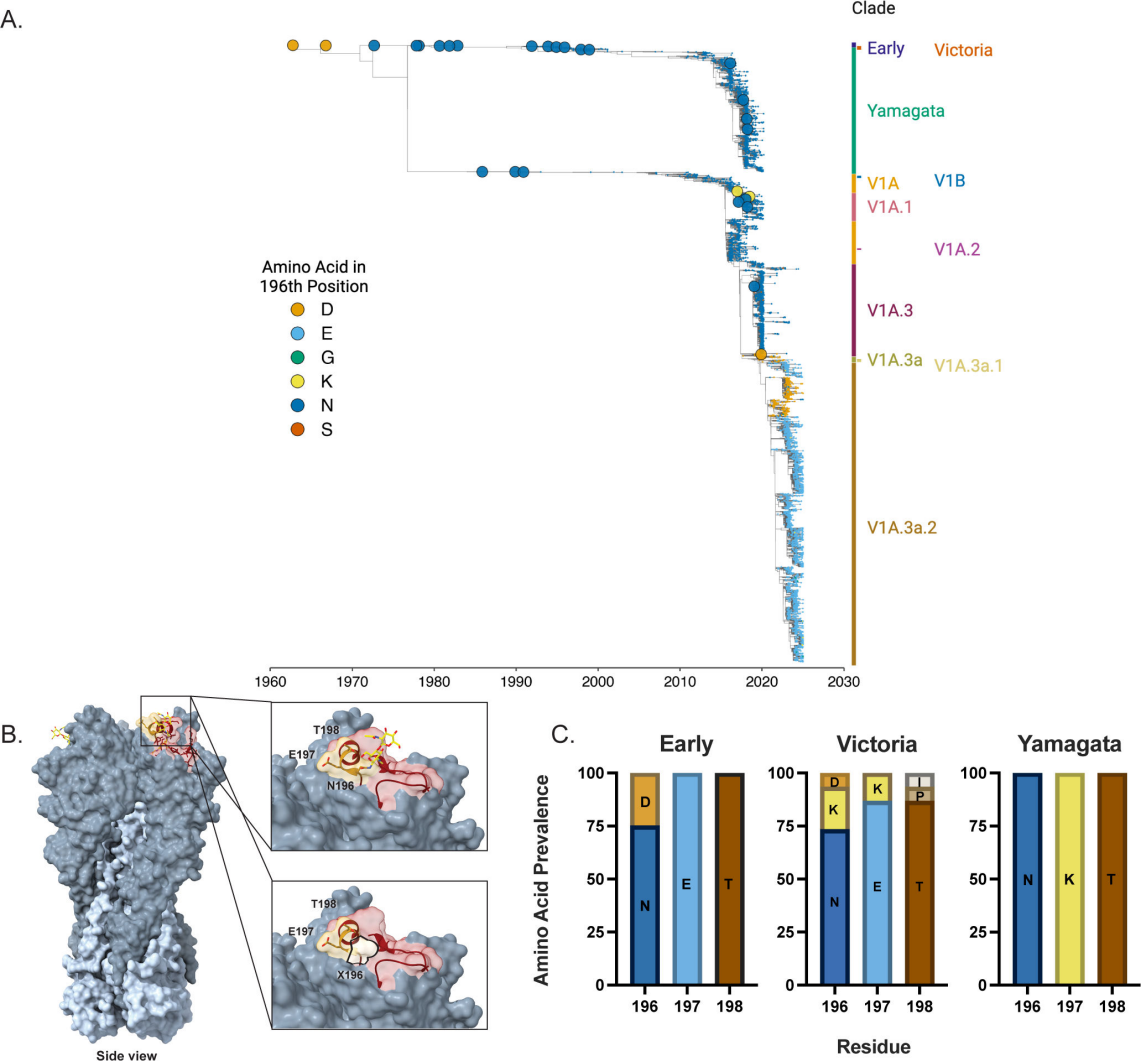
Phylogenetic analysis of influenza B viruses. (**A**) Phylogenetic tree of original or cell-passaged FLUBs HA sequences isolated between 1960 and 2024 represents the evolutionary relationships between Early (purple), Victoria (orange), and Yamagata (green) lineages. Mutations at residue N196 are color-coded to indicate the observed amino acid changes (D, E, G, K, N, S). Circles represent the strains selected for glycan microarray analysis in this study. (**B**) Diagram illustrating the glycosylation site within the receptor-binding domain and its alteration due to amino acid substitutions. The side view highlights the HA1 (dark gray) and HA2 (light blue) subunits and the receptor binding site (maroon). The upper shows an N-glycan (yellow) attached to the HA when the N-X-T motif is preserved (gold). The lower demonstrates the loss of the glycan when the asparagine (N) in the motif is mutated to another amino acid (X), disrupting the N-X-T glycosylation consensus sequence (PDB:4FQM). (**C**) Representation of amino acid mutations within the glycosylation site for the 29 viruses analyzed in this study. The data is categorized into Early (1962–1974; *n* = 4), Victoria lineage (1977–2019; *n* = 15), and Yamagata lineage (1991–2018; *n* = 10) groups.

The phylogenetic analysis of original or cell-passaged FLUBV HA sequences isolated between 1960 and 2024 revealed distinct evolutionary N-glycosylation patterns at residue 196 between the Early, Victoria, and Yamagata lineage viruses (Fig. 1A). While asparagine is the dominant residue at position 196 for both Victoria and Yamagata lineages, mutations at this site are more frequently observed in Victoria lineage sequences (Fig. S1). Mutations within the RBS at position 196 and its flanking residues can abolish the N-glycosylation sequon (N-X-S/T), thereby preventing the addition of an N-glycan (Fig. 1B). The appearance of specific variants (mutations N196D and N196E) is clustered closely with the emergence of successful contemporary subclades, such as V1A.3a.1 and V1A.3a.2, for the Victoria lineage (Fig. 1A). These subclades have been associated with increased endemic activity and have been isolated globally, including in East Asia, Southeast Asia, and North America (14). In contrast, substitutions at this position in the Yamagata lineage were observed less frequently, with no single variant rising to dominance.

Our data set of viruses used for glycan microarray analysis, indicated by circles in Fig. 1A, represents an antigenically diverse subset of FLUBVs, including Early viruses that circulated before the lineage split, as well as non-contemporary and contemporary Victoria and Yamagata lineage isolates (Table S1). Sequence analysis of the mammalian cell-propagated FLUBVs selected for microarray analysis revealed patterns consistent with the lineage differences in N-glycosylation at position 196 (Fig. S2), as observed in our phylogenetic analysis of the full data set (Fig. 1A). Our data showed that 25% (1/4) of Early viruses and 47% (7/15) of Victoria wild-type (WT) viruses had mutations at position 196, while all (10/10) Yamagata viruses retained the N-glycosylation motif (Fig. 1C; Fig. S2). Taken together, these data highlight distinct evolutionary glycosylation patterns at residue 196 for WT viruses from each lineage, independent of egg adaptations. Mutations at this site, potentially selected for due to their impact on viral fitness, may be contributing to the evolutionary success of recently emerged Victoria lineage subclades.

**Fig 2:**
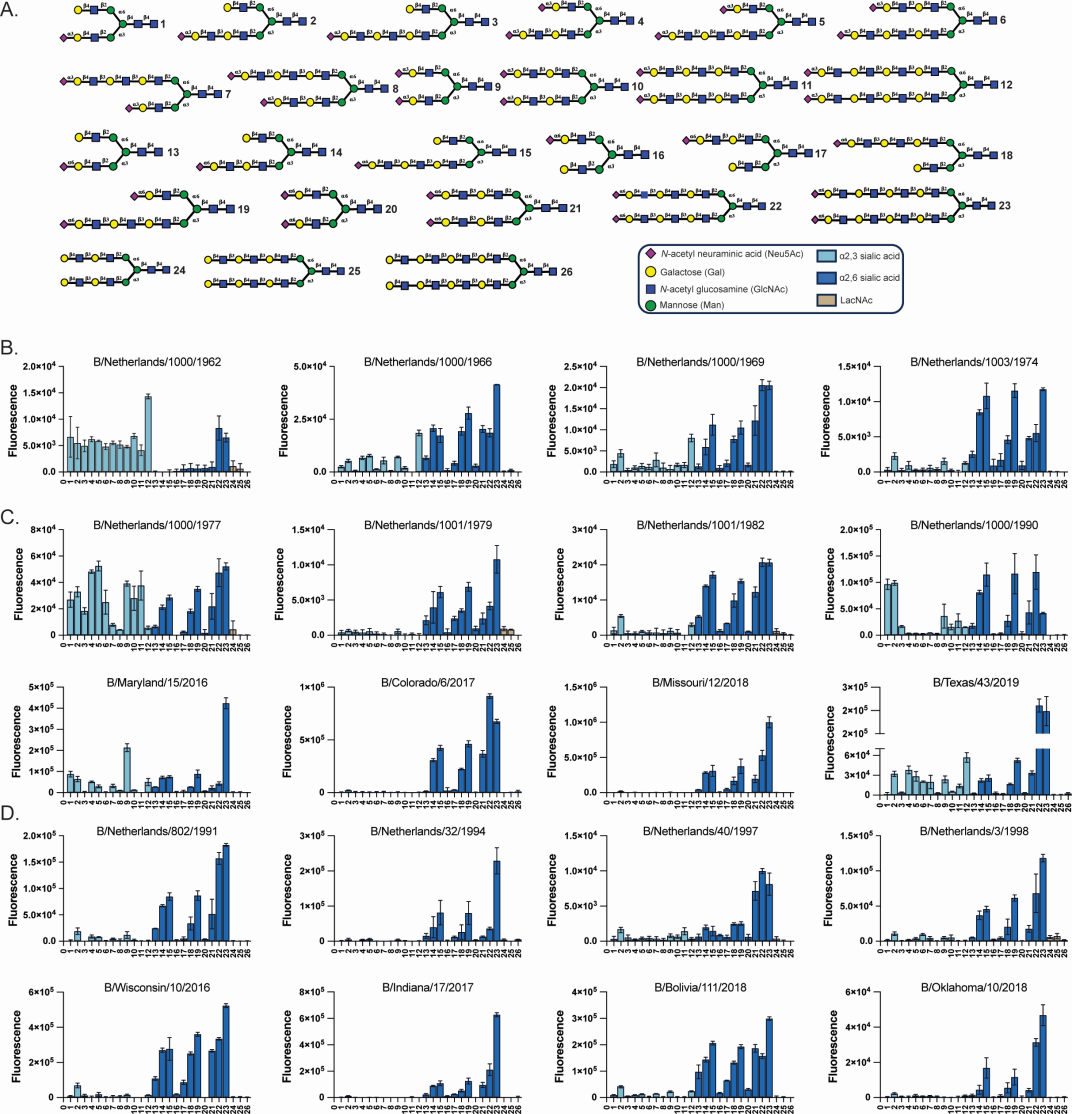
Glycan microarray analysis to define influenza B receptor specificity. (**A**) Structures of glycans printed on the microarray. Glycans 1–12 terminate with an α2,3-linked sialic acid, while glycans 13–23 terminate with an α2,6-linked sialic acid. Numbers correspond to the x-axis in B–D. (**B**) Microarray analysis of Early viruses. (**C**) Microarray analysis of Victoria lineage viruses. (**D**) Microarray analysis of Yamagata lineage viruses. Bars represent the average relative fluorescence units of four replicates ‡ SD.

### Receptor binding profiles of FLUBVs using glycan array technology

Most data regarding FLUBV receptor-binding profiles originated from isolates limited to small geographic regions, with minimal representation across evolutionary clades, leaving gaps in the comprehensive evolutionary characterization of the receptor-binding profiles of FLUBVs. Additionally, recent studies highlighted that, for FLUAV, it is not only the terminal sialic acid but also the structural elements of the glycan that influence binding (36–38). However, the significance of these factors for FLUBV remains undetermined.

To address this gap, we collected a globally diverse set of FLUBVs spanning 57 years, exclusively propagated in mammalian cell culture, and assessed their receptor-binding profiles using a glycan microarray designed to replicate structures found in the human respiratory tract (39). The microarray included symmetrical and asymmetrical biantennary N-glycans with multiple N-acetyl lactosamine (LacNAc) repeating units, capped by either α2,3- or α2,6-sialosides. Glycans are categorized by terminal modifications: α2,3-sialosides (1–12, light blue bars), α2,6-sialosides (13–23, dark blue bars), or unmodified galactose-terminating structures (24–26, tan bars) (Fig. 2A).

Cell-cultured virus isolates were applied to the microarray and detected using a human FLUBV HA stem monoclonal antibody (5E04) and a goat anti-human IgG antibody labeled with AlexaFluor-647 (40). The assay was performed in the presence of oseltamivir (OC), a neuraminidase (NA) inhibitor, to avoid interactions between the enzymatic protein and the glycans. The major binding differences observed between Early, Victoria, and Yamagata viruses lie in their ability to accommodate binding to α2,3-sialosides. Notably, only select Early and Victoria isolates exhibited this binding capability, while none of the Yamagata viruses did. Our earliest FLUBV, B/Netherlands/1000/62, along with an early Victoria strain, B/Netherlands/1000/1977, and more contemporary Victoria strains, B/Santiago/51375/2018 and B/Texas/43/2019, exhibited promiscuous binding to α2,3-sialosides and α2,6-sialosides, with varying mono-, di-, and tri-LacNAc repeating units (Fig. 2B and C; Fig. S3A). Other α2,3-sialoside-binding viruses, such as B/Maryland/15/2016, selectively bound compound 9, which is a symmetric bi-sialylated mono-LacNAc moiety, whereas the less contemporary B/Netherlands/1000/1990 preferentially bound compounds 1 and 2, which are asymmetric mono-sialylated α1,3-antenna mono- and di-LacNAc moieties, respectively.

Viruses isolated after 1962 consistently bound to α2,6-sialosides, with the strongest responsiveness to the extended symmetric bi-antennary compounds 22 and 23, which represent tri- and tetra-LacNAc moieties at the α1,3- and α1,6-antenna (bottom and top arms, respectively). Across lineages and clades, for α2,6-sialosides, we observed preferential binding to asymmetric di- and tri-LacNAc moieties compared to mono-LacNAc moieties (compounds 14, 15 and 18, 19 vs 13 and 16). Interestingly, reduced binding was observed when the Neu5Ac was presented at the α1,6-antenna compared to the α1,3-antenna. For example, viruses that bound to the asymmetric mono-LacNAc moiety, such as B/Bolivia/111/2018, lost recognition for this structure if the Neu5Ac was presented at the α1,6-antenna instead of the α1,3-antenna (13 vs 16). A similar pattern was observed with longer di- and tri-LacNAc isomeric structures, where viruses showed stronger responsiveness to compound 14 vs 17 and 15 vs 18, reinforcing the preference for the Neu5Ac presented on the α1,3-antenna. Of the asymmetric α2,6-sialosides, FLUBV’s showed the strongest binding to compounds 15 and 19, which represent a mono-sialylated α1,3-antenna tri-LacNAc and a bi-sialylated α1,3-antenna tri-LacNAc, respectively, highlighting the overall binding preference for longer glycan structures.

Sequence analysis revealed that viruses with dual binding capabilities frequently had mutations at or near the glycosylation site at position 196 (Table 1). No other shared mutations were identified that could be associated with the expansion of receptor specificity (Fig. S2). This suggested that the N-glycosylation site at position 196 may be a critical molecular determinant of receptor specificity and could drive lineage-specific binding differences in FLUBVs.

**TABLE 1:** Summary of viral N-glycosylation motifs and receptor binding specificity^*a*^

Virus strain	Lineage	196	197	198	α2,3	α2,6
**B/Netherlands/1000/1962**	**Early**	**D**	**E**	**T**	**+**	**+**
B/Netherlands/1000/1966	Early	N	E	T	+	+
B/Netherlands/1000/1969	Early	N	E	T	+	+
B/Netherlands/1003/1974	Early	N	E	T	−	+
**B/Netherlands/1000/1977**	**Victoria**	**N**	**E**	**I**	**+**	**+**
B/Netherlands/1001/1979	Victoria	N	K	T	−	+
B/Netherlands/223/1981	Victoria	N	K	T	−	+
B/Netherlands/1001/1982	Victoria	N	E	T	−	+
B/Netherlands/353/1985	Victoria	N	E	T	−	+
B/Netherlands/1000/1989	Victoria	N	E	T	−	+
**B/Netherlands/1000/1990**	**Victoria**	**N**	**E**	**P**	**+**	**+**
**B/Maryland/15/2016**	**Victoria**	**K**	**E**	**T**	**+**	**+**
B/Colorado/6/2017	Victoria	N	E	T	−	+
**B/Hong Kong/286/2017**	**Victoria**	**K**	**E**	**T**	**+**	**+**
B/Missouri/12/2018	Victoria	N	E	T	−	+
**B/Santiago/51375/2018**	**Victoria**	**K**	**E**	**T**	**+**	**+**
B/Hawaii/01/2018	Victoria	N	E	T	−	+
**B/Texas/43/2019**	**Victoria**	**D**	**E**	**T**	**+**	**+**
B/Washington/02/2019	Victoria	N	E	T	+	+
B/Netherlands/802/1991	Yamagata	N	K	T	−	+
B/Netherlands/25/1993	Yamagata	N	K	T	−	+
B/Netherlands/32/1994	Yamagata	N	K	T	−	+
B/Netherlands/8/1995	Yamagata	N	K	T	−	+
B/Netherlands/40/1997	Yamagata	N	K	T	−	+
B/Netherlands/3/1998	Yamagata	N	K	T	−	+
B/Wisconsin/10/2016	Yamagata	N	K	T	−	+
B/Indiana/17/2017	Yamagata	N	K	T	−	+
B/Oklahoma/10/2018	Yamagata	N	K	T	−	+
B/Bolivia/111/2018	Yamagata	N	K	T	−	+

^*a*^ This table lists the viruses analyzed, their lineage, their N-glycosylation motif amino acid sequences, and their receptor binding capability. Bolded viruses represent those which have mutations at or near the N-glycosylation motif and show dual binding profiles.

### Loss of glycosylation site broadens receptor specificity

After characterizing FLUBV receptor-binding patterns and identifying a potential role for the glycosylation site at position 196 (Table 1), we sought to investigate its impact on receptor binding in more detail. Due to its proximity to the RBS, the loss of this specific glycosylation motif may have exposed the receptor-binding pocket, enabling interactions with α2,3-sialosides in addition to α2,6-sialosides (34). Sequence analysis and binding assays following serial passage of WT FLUBVs in eggs suggested an expanded binding profile associated with mutations at this site (28, 29, 31, 32, 41). However, the direct contribution of HA residue 196 to receptor-binding specificity in WT viruses remained to be experimentally validated.

**Fig 3:**
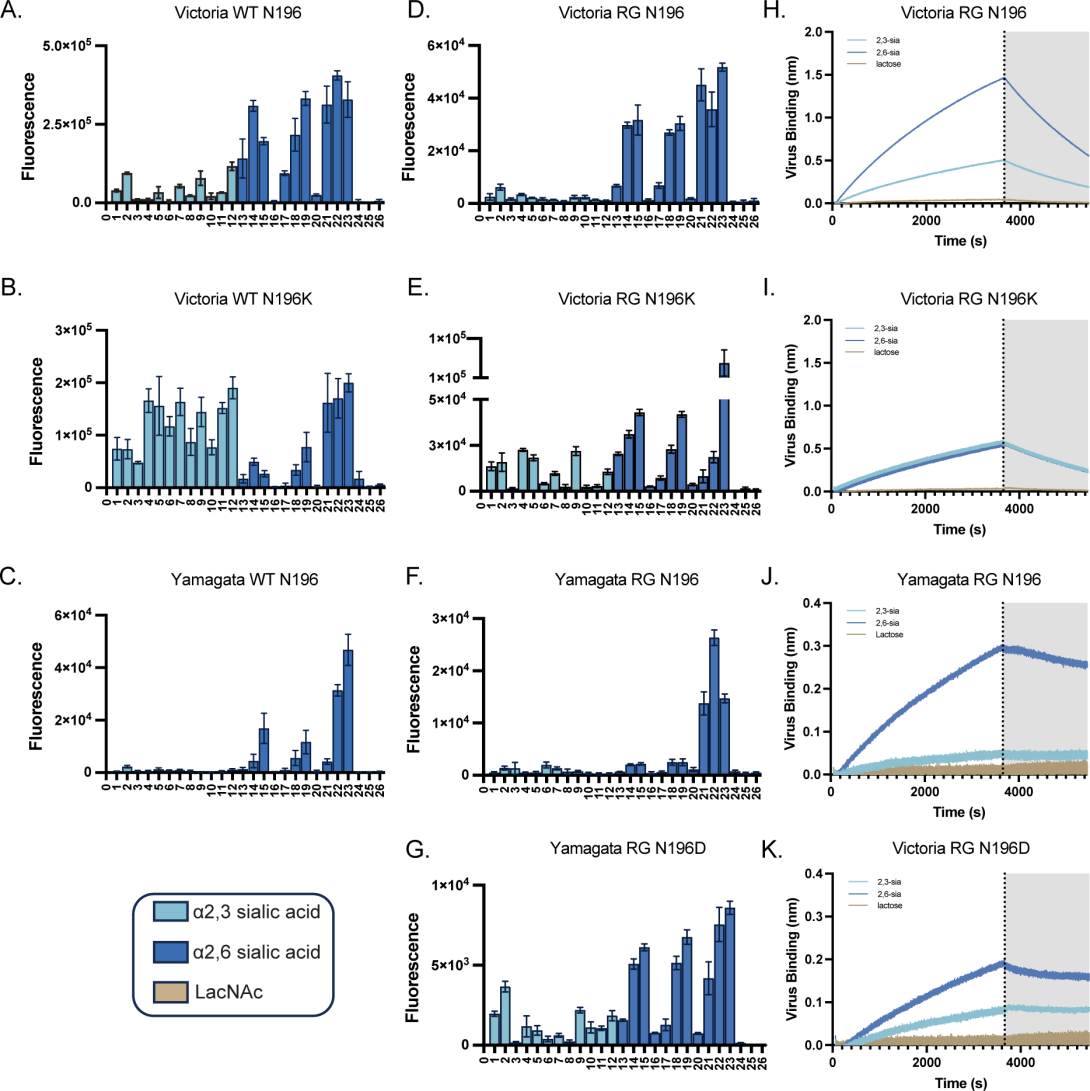
Receptor binding profiles of wild-type and reverse genetic viruses. (**A–C**) Represent the microarray analysis of wild-type (WT) viruses B/Hawaii/01/2018 (Victoria WT N196), B/Santiago/51375/2018 (Victoria WT N196K), and B/Oklahoma/10/2018 (Yamagata WT N196). (**D–G**) represent the microarray analysis of the reverse genetic (RG) viruses on a B/Brisbane/60/2008 backbone with the HA based on WT sequences of B/Hawaii/01/2018 (Victoria RG N196), B/Santiago/51375/2018 (Victoria RG N196K), B/Oklahoma/10/2018 (Yamagata RG N196), and the Yamagata mutant B/Oklahoma/10/2019 (Yamagata RG N196D). Bars represent the average relative fluorescence units of four replicates ‡ SD. (**H–K**) Biolayer interferometry (BLI) kinetics of the RG viruses binding to polyacrylamide (PAA) polymers capped with α2,3- or α2,6-sialyl-LacNAc or lactose.

We first examined the receptor-binding profiles of WT viruses (Fig. 3A through C). B/Hawaii/01/2018 (Victoria WT N196) naturally retained the 196 N-glycosylation site and preferentially bound α2,6-sialosides, with minimal α2,3 binding (Fig. 3A). In contrast, B/Santiago/51375/2018 (Victoria WT N196K) contained a naturally occurring mutation at position 196 and bound promiscuously to both α2,3- and α2,6-sialosides of varying LacNAc lengths and structures (Fig. 3B). Due to the rarity of mutations at the 196-glycosylation site in WT Yamagata viruses, B/Oklahoma/10/2018 (Yamagata WT N196) was the only WT virus used to represent this lineage. This isolate preferentially bound extended symmetric bi-antennary compounds 22 and 23 with responsiveness to asymmetric compounds 15 and 19 (Fig. 3C). These WT data demonstrated that natural variation at position 196 can correlate with distinct receptor-binding profiles, but other differences in the remaining six gene segments could also contribute.

To isolate the specific effect of HA residue 196, we generated reverse genetics (RG) viruses on a B/Brisbane/60/2008 backbone, including Victoria RG N196, Victoria RG N196K, Yamagata RG N196, and Yamagata RG N196D (mutation-induced variant not found in WT viruses) (42, 43). Figure 3D through G showed that the RG viruses reproduced the binding differences observed in the corresponding WT viruses, confirming that HA residue 196 is the primary determinant of receptor-binding specificity. The RG approach allowed for isolation of the specific role of mutations at the 196 N-glycosylation site in determining receptor specificity by eliminating influence from other gene segments, such as NA, which has an impact on receptor binding of some FLUAVs (44).

We further assessed real-time virus–glycan interactions using biolayer interferometry (BLI), a label-free technology. Streptavidin-coated BLI sensors were loaded with biotinylated polyacrylamide (PAA) polymers capped with α2,3- or α2,6-sialyl-LacNAc or lactose. Loaded sensors were then dipped into a buffer containing the virus of interest to allow HA binding. Oseltamivir was included to block NA activity. Our Victoria RG N196 virus bound strongly to α2,6-sialyl-LacNAc, with minimal α2,3 binding (Fig. 3H), while mutating the glycosylation site led to equal binding with both α2,3 and α2,6 polymers (Fig. 3I). Similarly, Yamagata RG N196 showed strong α2,6 binding, with α2,3 binding comparable to the negative lactose (Fig. 3J). Notably, the Yamagata RG N196D virus exhibited increased binding to α2,3-sialyl-LacNAc, compared to the Yamagata RG N196 virus, while maintaining the α2,6 binding (Fig. 3K). These findings highlight the significant role of single amino acid mutations that disrupt the 196 N-glycosylation motifs in influencing influenza B glycan-virus interactions and shaping receptor-binding properties.

### Molecular dynamics simulations reveal molecular origin of glycan-mediated receptor specificity

**Fig 4:**
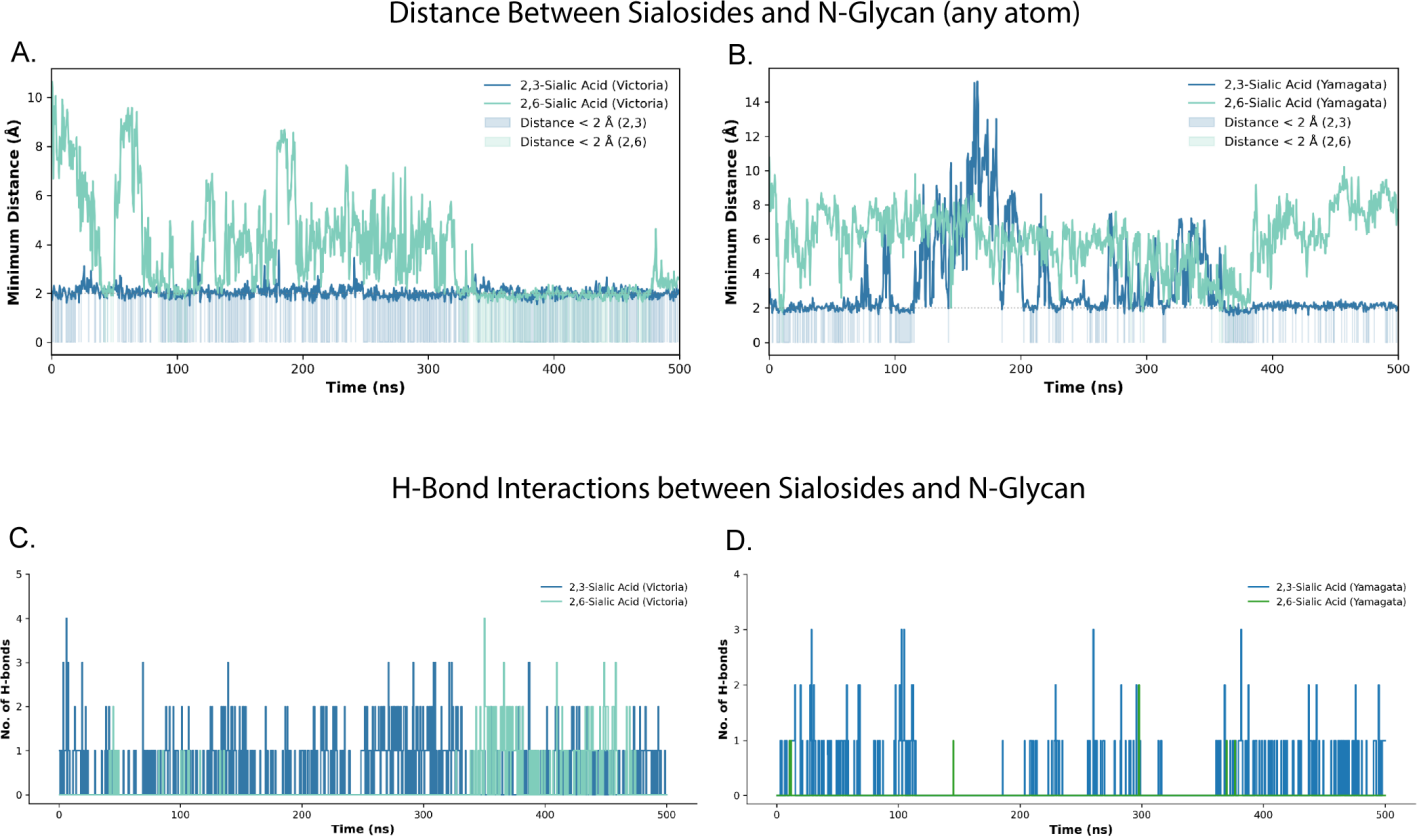
N-Glycan interactions with sialosides in Victoria vs Yamagata (**A–B**) Minimum distances (any atom) between the N-glycan and α2,3-linked (blue) or α2,6-linked (teal/green) sialic acids for Victoria (**A**) and Yamagata (**B**). (**C–D**) Comparative analysis of hydrogen bonding between N-glycan and α2,3-/α2,6-sialosides across (**C**) Victoria and (**D**) Yamagata influenza lineages over 500 ns MD simulations.

Building on experimental data, we performed molecular dynamics (MD) simulations to further explore the role of the 196 N-glycan in receptor binding of FLUBVs with α2,3- and α2,6-sialosides. The HA trimer structures of FLUBV B/Hawaii/01/2018 and B/Oklahoma/10/2018 were modeled using AlphaFold3 (45), with point mutations at residue 196 (N196K and N196D, respectively) introduced using the Rosetta software suite (46). Sialoside receptor analog comprising a linear pentasaccharide with a terminal α2,3-linked Neu5Ac was modeled using the GLYCAM-Web server (47), and the 2,6-linked conformation was derived from the H3N2 crystal structure (PDB: 6AOV). The confidence of AlphaFold3-predicted structures was assessed using the per-residue confidence metric (pLDDT). Both B/Hawaii/01/2018 (Victoria) and B/Oklahoma/10/2018 (Yamagata) HAs displayed high pLDDT scores, with most residues scoring above 90 for the RBS (Fig. S4). MD simulations were performed on Victoria (B/Hawaii/01/2018) and Yamagata (B/Oklahoma/10/2018) HA trimers in complex with α2,3- and α2,6-linked sialosides, with an N-glycan attached at residue 196. Proximity analysis of sialoside atoms and N-glycan atoms at position N196 revealed distinct interaction patterns. The α2,3-linked sialosides consistently approached the N-glycan at closer distances compared to α2,6-linked sialosides, often entering the hydrogen-bond (H-bond) interaction zone, suggesting stronger interactions between α2,3-linked sialosides and N-glycan for both the Yamagata and Victoria simulations (Fig. 4A and B). Analysis of the H-bond interactions between sialosides and the N-glycan revealed striking differences between α2,3- and α2,6-linked sialosides. For both Victoria and Yamagata lineage viruses, α2,6-linked sialosides rarely formed H-bonds with the N-glycan, whereas α2,3-linked sialosides formed multiple H-bonds consistently with N-glycan over the 500 ns simulation period (Fig. 4C and D).

**Fig 5:**
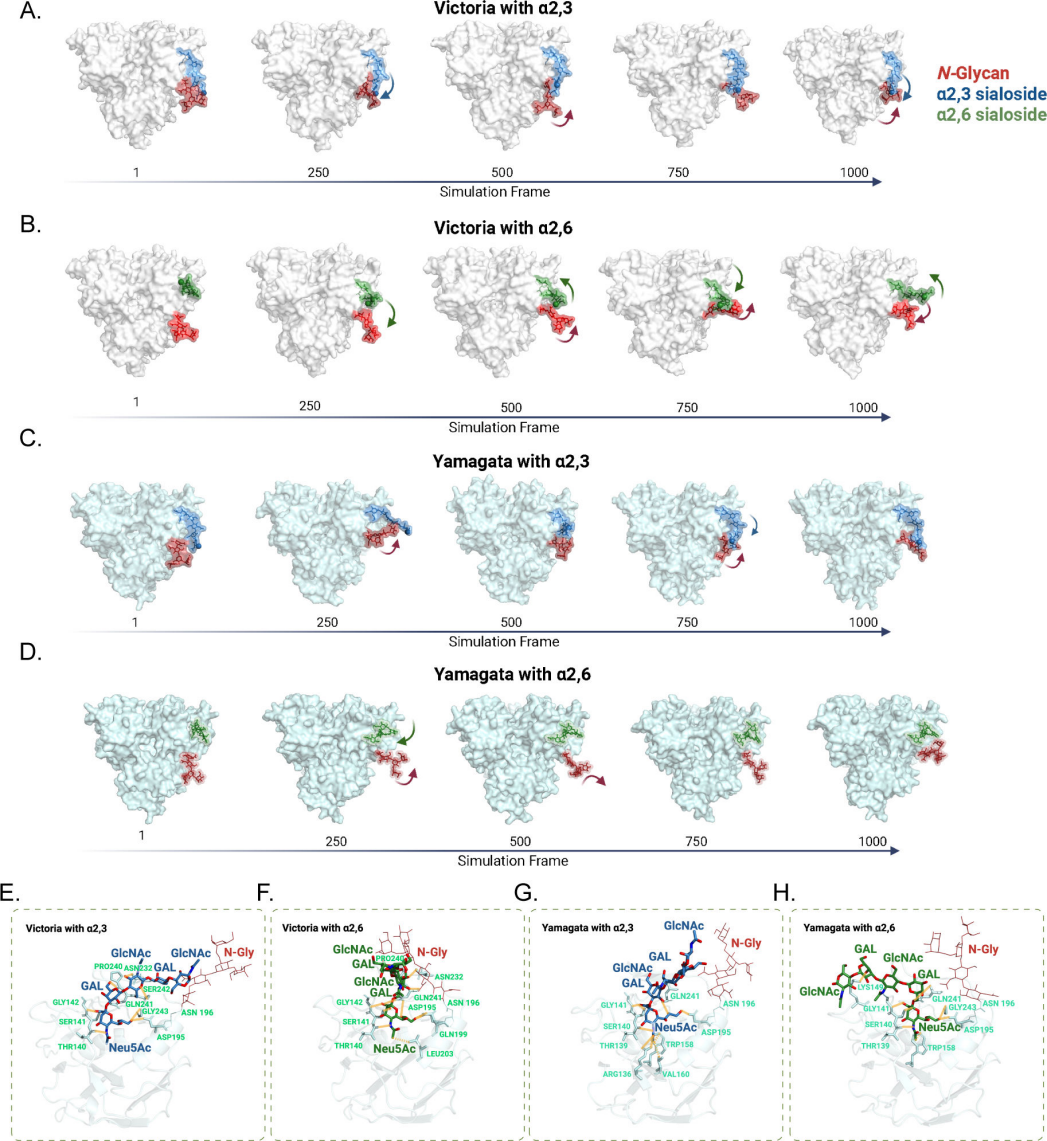
Structural interactions of HA with α2,3- and α2,6-linked sialic acids. (**A–D**) Snapshots of molecular dynamics trajectories showing the positions of α2,3-linked (blue) and α2,6-linked (green) sialosides relative to the N-glycan (red) over simulation frames. (**A–B**) Victoria hemagglutinin with α2,3- and α2,6-linked sialosides, respectively. (**C–D**) Yamagata hemagglutinin with α2,3- and α2,6-linked sialosides, respectively. Simulation frames are labeled along the timeline (1 to 1,000). (**E–H**) Shows the structural interactions of the sialic acids and N-glycan with the HA. The sialic acid backbone (SIA), galactose (GAL), and N-acetylglucosamine (GINAc) are labeled for clarity. Dotted lines represent hydrophobic interactions, whereas solid lines represent hydrogen bonds.

This phenomenon was further supported by representative MD snapshots (Fig. 5A through D). In the Victoria α2,3 simulation (Fig. 5A), the sialoside mostly occupied conformations within close distances to the N-glycan, indicating persistent favorable interactions with the N-glycan. A similar trend emerged for the Yamagata α2,3 simulation (Fig. 5C), where the sialoside remained in close contact with the N-glycan. By contrast, α2,6-linked sialosides in Victoria (Fig. 5B) and Yamagata (Fig. 5D) simulations generally maintained a greater distance than α2,3-linked sialosides.

### Structural interactions between sialosides and HA altered by the presence of N-glycan

To obtain structural insight into how sialoside linkage type modulates glycan binding in Victoria and Yamagata lineage viruses, we extracted a representative snapshot from 1,000 molecular dynamics frames by principal component analysis (PCA) (Fig. S5). In the Victoria-α2,3 complex, 140-loop residues Thr140, Ser141, Gly142, together with 240-loop residues Gln241 and Gly243, form hydrogen bonds to Neu5Ac. This replicates the binding pattern of B/HK HA–LSTa crystal (2RFT), where Neu5Ac made multiple hydrogen bonds with these 140-loop residues (Thr140, Ser141, Gly142) (19). The proximal Gal also hydrogen-bonds to the backbone carbonyl near Pro240, and proximal GlcNAc forms hydrogen-bonds with Ser242, which showed the same exact interactions that were observed for B/HK HA–LSTa crystal complex (2RFT) (19).

The Yamagata-α2,3 complex with the intact N-glycan at Asn196 showed almost similar interaction patterns as the Victoria-α2,3 complex, where Neu5Ac is stabilized by Thr139, Ser140, Gly141, and Asp195, while the distal GlcNAc remains sterically blocked by the N-glycan. Notably, in Yamagata, Trp158–Neu5Ac hydrophobic packing is evident. For the human α2,6 sialopentasaccharide (LSTc), our models from representative 500-ns MD frame show that Sia-1 is anchored by multiple hydrogen bonds to the 140-loop-backbone N–H and C=O of Gly142, the side-chain hydroxyl of Ser141, and the backbone carbonyl of Thr140, with additional contacts from the Neu5Ac to Asp195, consistent with the B/HK HA–LSTc crystal (19). Although the crystal 2RFU exhibits fragmented density and limited asialo contacts, we observe stable distal interactions in the complexes, including a recurrent distal-Gal and Lys149 hydrogen bond (Yamagata) and a reproducible Gal and backbone-carbonyl interaction at residue Pro240 (Victoria), in line with the weak Gal contact noted in the HA–LSTc crystal complex 2RFU (19). For all the sialopentasaccharides, our models and the previous co-crystals (2RFT, 2RFU) indicate engagement beyond Sia-1 from the nearer Gal, the adjacent GlcNAc, and the distal Glc.

Hydrogen-bond network analysis showed that, in the glycosylated state (N196 present), α2,6-linked sialosides form a markedly denser and more extensive hydrogen-bond network than α2,3-linked sialosides in both Victoria and Yamagata HAs. Disruption of the N196 glycosylation site (Victoria N196K; Yamagata N196D) abolishes this disparity, yielding convergent, similarly distributed hydrogen-bonding patterns for α2,3 and α2,6 sialosides (Fig. S6).

Root-mean-square deviations (RMSD) calculation for backbone atoms showed that all systems reached equilibrium at early stages and remained stable over 500 ns, with backbone RMSD values ranging within a few Å (Fig. S7). Root-mean-square fluctuation (RMSF) analysis of HA backbone atoms revealed no significant differences in flexibility between α2,3- and α2,6-linked sialosides (Fig. S8), including the RBS residues, suggesting that the divergent binding preferences are driven by local interaction effects, such as N-glycan-mediated interactions with α2,3 sialosides, rather than global changes in HA dynamics.

## DISCUSSION

In this study, we comprehensively characterized receptor binding across Early, Victoria, and Yamagata lineage FLUBVs, including contemporary strains, using glycan arrays that mimic structures found in the human respiratory tract. Historically, influenza receptor binding was thought to be determined primarily by the terminal sialic acid; however, recent studies have shown that the structural topology of the glycan itself also plays a critical role in receptor binding (37, 38, 48, 49). Our binding studies with symmetrical and asymmetrical N-glycans revealed that, in addition to the length of the LacNAc structure, the presentation of Neu5Ac on a specific antenna influenced binding. FLUBV’s across lineages and clades preferentially bind extended symmetric bi-antennary compounds sialylated at the α1,3 and α1,6-antennas. These viruses also showed greater responsiveness to α2,6-linked sialosides presented on extended LacNac structures at the α1,3-antenna. In contrast, shorter mono- and di-LacNac structures abolished binding when the epitope was presented on the α1,6-antenna, showing a preference for the α1,3-antenna. This preference aligns with the activity of human sialyltransferase ST6Gal1, which preferentially modifies the α1,3-antenna over the α1,6-antenna on N-linked glycans, indicating an adaptation of FLUBVs to human hosts (40, 50). The primary difference between lineages was the expansion of binding profiles in some Early and Victoria lineage viruses. Our findings supported previous research showing that Yamagata viruses primarily bound α2,6-linked sialosides, while Victoria viruses preferentially bound α2,6 but can also adapt to bind α2,3-sialosides. Additionally, early FLUBVs share similar binding preferences to Victoria viruses, with some viruses able to expand from binding only α2,6-sialosides to also bind α2,3-sialosides. Given the closer evolutionary relationship between Early and Victoria viruses, it follows that they exhibit similar binding patterns.

For FLUAVs, the importance of receptor specificity is well-defined, with human viruses binding α2,6-sialosides and avian viruses binding α2,3-sialosides. Mutations within the RBS have been shown to broaden the binding capacity of avian viruses, enabling them to recognize α2,6-sialosides and thereby facilitating interspecies transmission (33, 51, 52). The importance of receptor specificity for FLUBVs is more nuanced, as some Early and Victoria lineage viruses can bind both α2,6- and α2,3-sialosides, yet these viruses remain restricted to human hosts. This limitation is partly due to FLUBVs' long-standing adaptation to human hosts, which requires specific temperature and pH conditions that are found in the human upper respiratory tract (53). For FLUBVs, receptor specificity may contribute to the observed differences in age-related host susceptibility between the lineages: Victoria viruses typically infect younger children, who have a higher prevalence of α2,3-linked sialosides in their upper respiratory tract, while Yamagata viruses predominantly infect older populations with increased expression of α2,6-linked sialosides in their upper respiratory tract (1, 34, 54–57). Additionally, dual sialoside-binding FLUBV viruses have been associated with increased disease severity by causing gastrointestinal illness and lower lung infections, likely due to the higher proportion of α2,3-linked sialosides in these mucosal tissues, enabling disseminated infection (18, 56–58). While we do not suggest that the broadened receptor specificity of Early and Victoria viruses facilitates cross-species transmission, it is important to consider the broader host implications.

Molecular determinants influencing receptor specificity for FLUBVs remain largely undefined. Sequence analysis of the cell-cultured FLUBVs used in the glycan array studies revealed mutations at the 196 N-glycosylation site in Early and Victoria viruses, which resulted in expanded binding profiles from α2,6-sialosides to also include α2,3-sialosides (Table 1). These findings suggested that this mutation may be a key determinant of receptor specificity in FLUBVs. Previous structural modeling (19) based on FLUBV HA sequences suggested that N-glycosylation within the RBS may influence receptor binding by physically obstructing the binding pocket (34). Our experimental data support this hypothesis: the loss of the 196 N-glycosylation site broadens receptor specificity, as shown using a glycan microarray (Table 1; Fig. 2; Fig. S3). This expanded specificity was further validated using the FLUBV RG system, where RG-generated viruses from both lineages with the mutated N-glycosylation site bound both α2,3- and α2,6-sialosides, whereas RG viruses retaining the N-X-S/T glycosylation motif bound exclusively to α2,6-sialosides. These results underscore the critical role of this N-glycosylation site in modulating FLUBV receptor binding, enabling interaction with both α2,3- and α2,6-linked sialic acids independent of lineage (Fig. 3). While other distinct glycosylation patterns exist, such as the functional glycosylation site at position 233 found only in Victoria lineage viruses (34), the distal location of this glycan from the RBS makes it less likely to play a role in modulating receptor specificity differences between the lineages. For FLUAV, the balance between receptor-binding HA and the enzymatically active NA is known to significantly impact receptor binding (59). Although we did not explicitly investigate HA:NA balance in FLUBVs, our observation that RG viruses maintained consistent binding profiles regardless of gene segment swaps suggests that HA:NA balance may play a less critical role in the more conserved FLUBVs. Further studies are needed to confirm this hypothesis and explore its implications.

Our results highlighted distinct evolutionary mechanisms between the FLUBV lineages, with Victoria viruses exhibiting greater variability at the 196-glycosylation site, allowing for mutations that abolish the glycosylation motif. In contrast, such mutations are rarely observed in Yamagata viruses (34). We suggest that Victoria viruses have selectively favored these mutations, as shown by our phylogenetic analysis, which indicated that variants with N196 mutations and therefore, broadened sialoside binding, are clustered with successful contemporary subclades, such as V1A.3a.1 and V1A.3a.2. These subclades dominated the post-2021 influenza seasons and were subsequently included in the annual influenza vaccine formulation. In contrast, while isolated N196 mutations occasionally occurred in Yamagata viruses, no single variant has achieved dominance, underscoring the different evolutionary strategies of the two lineages. The limited acquisition of N196 mutations in Yamagata, combined with widespread COVID-19 mitigation measures, such as masking, social distancing, and reduced travel, may have contributed to the disappearance of this lineage from global circulation. Interestingly, our findings showed that while wild-type Yamagata viruses infrequently acquire this mutation, they can accommodate the change, and like the Victoria lineage, it expanded their ability to bind α2,3-linked sialosides. This adaptation could have implications for the tropism and fitness of Yamagata viruses, potentially increasing susceptibility in younger populations or contributing to more severe symptoms associated with infection.

Our study also provides insights into the structural role of the 196 N-glycan on receptor specificity by integrating molecular modeling, site-directed mutagenesis, and MD simulations. The presence of this N-glycan creates a pronounced difference in interactions that allows for binding of α2,3-linked sialosides with N glycan, while disfavoring binding of α2,6-linked sialosides in both Victoria and Yamagata lineages (Fig. 5). Notably, when interacting with FLUBV HA, the α2,3-linked sialosides adopt a *trans* conformation, similar to observations with other influenza viruses like avian H5 and avian H3 (19, 33, 60–62). This restricted their flexibility and directed the α2,3-linked sialosides to interact with the N-glycan, which results in stable hydrogen-bond formation with the N-glycan instead of the RBS alpha helix region. In contrast, α2,6-linked sialosides predominantly assumed a *cis* conformation with enhanced conformational freedom, allowing them to escape interaction with N-glycan and achieve extended engagement with RBS. This Neu5Ac conformation-dependent differential interaction emerged as a major determinant of receptor specificity for FLUBVs (19). The hydrogen-bond network analysis identified interactions between the Neu5Ac and conserved Thr-140, Ser-141, and Gly-142, along with various other residues, corroborating our model while building off previous structural analysis. Our 500-ns MD for the α2,6-linked LSTc aligns with crystal complex 2RFU: Sia-1 is anchored by the 140-loop (Thr140/Ser141/Gly142), matching the crystal geometry. For the α2,3-linked LSTa, our complexes align the HA–LSTa crystal complex 2RFT where besides Neu5Ac both proximal and distal Gal and GlcNAc make interactions with receptor binding site residues. These findings underscore the regulatory influence of glycosylation at N196 on receptor specificity, with potential ramifications for viral host age range and pathogenicity. Moreover, our MD simulations revealed that these local receptor-binding perturbations exert minimal impact on the global conformational landscape of the HA protein.

Despite the insights provided by our integrative modeling and simulation approach, several limitations should be noted. Our structural analyses relied on AlphaFold-predicted HA models with Rosetta-introduced mutations, which, while supported by high local confidence scores, do not replace experimentally determined FLUBV HA–sialoside co-crystal structures. Similarly, the glycan conformations used in docking and MD simulations were derived from modeled or heterologous templates and may not capture the full conformational diversity of host receptors. Although 500 ns production MD simulations showed stable trajectories, longer timescales may be needed to sample slower rearrangements or rare binding events. All computational analyses depend on force fields, parameterization, and modeling assumptions, introducing additional uncertainty. Additionally, while our receptor binding analysis represents a significant improvement over what is currently available, it is heavily biased toward viruses obtained from the Netherlands. This geographic bias may limit the generalizability of our findings. Therefore, our conclusions from structural models, MD simulations, and the data set should be viewed as complementary to, rather than a substitute for, direct experimental validation and broader sampling.

In conclusion, this study provided crucial insights into the receptor specificity of FLUBVs, emphasizing the role of the N-glycosylation site at residue 196 in modulating binding profiles. Our findings highlighted how mutations at this site can expand receptor specificity, allowing for the recognition of both α2,6- and α2,3-linked sialosides. These molecular determinants offer a clear mechanism for lineage-specific differences in HA receptor binding, contributing to a deeper understanding of FLUBV evolution.

## MATERIALS AND METHODS

### Viruses

A collection of 16 influenza B viruses, exclusively passaged in mammalian cells, was provided by Dr. Ron Foucher’s lab (Table S1). Additional parental isolates were obtained from the International Reagent Resource (IRR), with passage history and IRR numbers listed in Table S1. Viral stocks were generated in Madin-Darby Canine Kidney (MDCK-ATL; FR-926) cells cultured in Dulbecco’s Modified Eagle Medium (DMEM) with 5% fetal bovine serum (FBS). Cells were inoculated with viral dilutions in DMEM without FBS and 1 µg/mL tosylsulfonyl phenylalanyl chloromethyl ketone (TPCK)-treated trypsin and incubated at 35°C with 5% CO₂ for 72 h. Supernatants were collected, aliquoted, and stored at −80°C. All HA sequences were confirmed by next-generation sequencing and are available in the GISAID EpiFlu database (Table S1). Reverse genetics was used to generate four influenza B viruses with an isogenic internal gene and NA background derived from B/Brisbane/60/2008, differing only in their HA (63). HA sequences were derived from B/Oklahoma/10/2018 (wild type), B/Oklahoma/10/2018 (N196D), B/Hawaii/01/2018, and B/Santiago/51375/2018. HA plasmids were synthesized by Twist Bioscience (San Francisco, CA) and cloned into the bidirectional pDP2002 vector (64). Virus generation was performed using a co-culture of HEK293T and MDCK cells seeded at a ratio of 6:1 and transfected with 1 µg of each of the eight plasmids (total 8 µg) using 18 µL of TransIT-LT1 transfection reagent (Mirus Bio LLC, Madison, WI). The transfection mixture was incubated for 45 min and then added to the cells. After overnight incubation, the transfection mixture was replaced with Opti-MEM media containing 1% antibiotic-antimycotic solution (Life Technologies, Carlsbad, CA). At 24 h post-transfection, the media were supplemented with 1 µg/mL of (TPCK)-treated trypsin (Worthington Biochemicals, Lakewood, NJ). Viral sequences were confirmed by Sanger sequencing, and viruses were propagated in MDCK cells to prepare viral stocks.

### Phylogenetic analysis

All hemagglutinin (HA) sequences and corresponding metadata were obtained from GISAID (65). HA residues are numbered according to the mature protein sequence of B/Brisbane/60/2008 (PDB: 6FYW, Victoria lineage) after cleavage of the signal peptide; position 196 corresponds to the major N-glycosylation site. Sequences spanning the years 1960–2024 were downloaded with the following inclusion criteria: human host, complete HA gene, and original cell-passaged sequences only. For sequences from 1960 to 2000, due to the unavailability of original-passaged complete HA sequences, we included cell-passaged sequences. Sequence alignment was performed using MAFFT (v7) (66), which was implemented in the Seqtron tool (67). Duplicate amino acid sequences were filtered, and the corresponding DNA sequences for the unique amino acid sequences were retained for downstream analyses (6,357 sequences). Maximum likelihood phylogenetic trees were generated using IQ-TREE (v2.2.2) (68) with the best-fit model TVM+F+R4. The tree was then used as input for TreeTime (v0.11) (69) to calibrate the phylogeny to a time scale. We used the *Baltic* Python package to map and visualize mutation information on the fixed maximum likelihood tree generated by IQ-TREE. We also analyzed all publicly available IBV sequences deposited in GISAID through 31 December 2024 (*n* = 78,052) and identified the presence or absence of the 196 N-glycosylation site.

### Microarray printing and binding analysis

All N-glycans bear an α-amine at the reducing end asparagine moiety and were printed on amine reactive, NHS-ester activated glass slides (NEXTERION Slide H, Schott Inc.) using a Scienion sciFLEXARRAYER S3 non-contact microarray equipped with a Scienion PDC80 nozzle (Scienion Inc.). Individual samples were dissolved in sodium phosphate buffer (50 µL, 0.225 M, pH 8.5) at a concentration of 100 µM and were printed in replicates of 6 with a spot volume of ~400 pl at 20°C and 50% humidity. Each slide has 24 subarrays in a 3 × 8 layout. After printing, slides were incubated in a humidity chamber for 8 h and then blocked for 0.5 h in a Tris buffer (pH 9.0, 50 mM) containing 5 mM ethanolamine at 50°C. Blocked slides were rinsed with DI water, spun dry, and kept in a desiccator at room temperature for future use.

Binding analysis was performed by incubating the slides with virus isolates at optimized dilution in TSM binding buffer ([TSM-BB]); Tris-HCl 20 mM pH 7.4, NaCl 150 mM, CaCl_2_ 2 mM, MgCl_2_ 2 mM, containing 1% BSA and 0.05% Tween-20) in the presence of a neuraminidase inhibitor (Oseltamivir carboxylate, OC, 10 µM) for 2 h at room temperature. Then, slides were washed by sequentially dipping in TSM wash buffer (2 min, containing 0.05% Tween 20), TSM buffer (2 min), and water (2 × 2 min), followed by centrifugation. Next, slides were incubated with a premixed solution of a FLUBV monoclonal antibody 321.05.05.PB.5E04 (0.1 µg/mL) and goat anti-human IgG antibody Alexa Fluor 647 (1 mg/mL) (Jackson ImmunoResearch) in TSM-BB in the presence of OC (10 µM) for 1 h, followed by washing and drying.

The slides were scanned using a GenePix 4000B microarray scanner (Molecular Devices) at the appropriate excitation wavelength with a resolution of 5 µm. Optimum gains and PMT values were employed for the scanning, ensuring that all signals were within the linear range of the scanner’s detector and that there was no saturation of signals. The images were analyzed using GenePix Pro 7 software (version 7.2.29.2, Molecular Devices). The data were analyzed with a home-written Excel macro. The highest and the lowest values of the total fluorescence intensity of the replicate spots were removed, and the remaining values were used to provide the mean value and standard deviation. The fluorescence values were plotted using Prism 10 software (GraphPad Software, Inc.); bars represent the mean ± SD for each treatment.

### Biolayer interferometry

BLI analysis was performed using an Octet RED384 system at 30°C. Streptavidin (SA) biosensors were loaded with biotinylated synthetic PAA polymers capped with either α2,3- or α2,6-sialyl-LacNAc or lactose at a concentration of 5 µg/mL for 600 s. WT and RG viruses were diluted to equivalent hemagglutinating units of 128 for Victoria lineage viruses and 32 for Yamagata lineage viruses in TSM-BB containing 0.1% BSA, 0.05% Tween-20, and 10 µM OC. Real-time virus association was monitored for 3,600 s by transferring the loaded sensors into 80 µL dilutions of virus. Dissociation was recorded by immediately moving the sensors into TSM-BB for 1,800 s. Data were analyzed using Octet Data Analysis software, and binding curves were generated in GraphPad Prism.

### Structural modeling and mutagenesis

Although IBV HA co-crystal structures exist (e.g., PDB 2RFT, 2RFU), our Victoria (B/Hawaii/01/2018) and Yamagata (B/Oklahoma/10/2018) sequences differ substantially from those templates, so we predicted the exact lineage HA trimers using AlphaFold3 (70). Point mutations at residue 196 were introduced using Rosetta (71): N196K for B/Hawaii/01/2018 and N196D for B/Oklahoma/10/2018. Each mutated structure underwent Cartesian relaxation in Rosetta to refine sidechain packing and backbone geometry. For receptor analogs, we focused on sialosides with five terminal sugar units: DNeup5Aca2-3DGalpb1-4DGlcpNAcb1-3DGalpb1-4DGlcpNAca1-OH for 2,3-linked sialic acid, and DNeup5Aca2-6DGalpb1-4DGlcpNAcb1-3DGalpb1-4DGlcpNAca1-OH for 2,6-linked sialic acid. Our assays used receptor analogs matching host receptors 3′SLN-β1-4 (α2-3) and 6′SLN-β1-4 (α2-6), but no IBV HA co-crystals contain these full five-sugar ligands (IBV crystal structures largely feature shorter α2-6 or α2-3 with β1-3 internal linkages). Accordingly, we extracted α2-6 sialoside from the H3 LSTc complex (PDB 6AOV), and for α2-3, we built the α2-3 3′SLN-β1-4 model with GLYCAM-Web server, then superposed the sialic-acid/Gal anchor onto our Alphafold3 IBV models and applied local minimization before MD (47). For HAs retaining the native N196 residue, a high-mannose type N-glycan (DManpa1-3[DManpa1-6]DManpb1-4DGlcpNAcb1-4DGlcpNAcb1-OH) was appended at the glycosylation site using the glycoprotein builder tool of the GLYCAM server (47). All sialic acid placements were guided by structural superposition of both the HA head receptor-binding domain (residues 71–271) and sialosides to the reference crystal structures (PDBs: 2RFT, 4YYA, and 6AOV) using PyMOL (72). Then those systems containing both protein and sialosides were exported and used as the initial structure for molecular dynamics simulation.

### Molecular dynamics simulations and analysis

Using AmberTools24 (73), a total of eight systems were prepared for MD simulations: four with native N196 glycans with N-glycan attached to the site, and four with N196K or N196D mutations, each bound to either 2,3- or 2,6-linked sialic acid. All the systems were then solvated in an octahedral box of TIP5P water models (74) and were neutralized with counter-ions. To ensure stable dynamics, a 10-step MD preparation protocol described by Roe and Brooks (75) was adopted. This protocol includes sequential energy minimizations and short MD relaxation steps with gradually reduced restraints, followed by a final NPT simulation until density stabilization. Using Amber24 (76), production runs of 500 ns at 300 K and constant pressure were conducted. For glycans and sialic acid, GLYCAM_06j-1 (77) and for HA protein ff19SB (78) force fields were used. Trajectories were analyzed using CPPTRAJ (79) for RMSD, RMSF, and hydrogen-bond networks. Besides, to identify the most representative conformation of the complex, PCA was done on 1,000 frames of the simulation trajectory, and the frame corresponding to the highest density in the projection was extracted for visualization and was examined with the Protein-Ligand Interaction Profiler server (80).

## Data Availability

Data supporting the findings of this study are available within the paper and supplemental material and from the corresponding authors upon reasonable request. Accession numbers for the sequences of virus HA and NA gene segments are listed in Table S1.

## SUPPLEMENTAL MATERIAL

Supplemental material jvi.01039-25-s0001.pdf Figures S1 to S8; Table S1.

ASM does not own the copyrights to Supplemental Material that may be linked to, or accessed through, an article. The authors have granted ASM a non-exclusive, world-wide license to publish the Supplemental Material files. Please contact the corresponding author directly for reuse.
