# Supplementary material for: N-glycosylation at the receptor binding site drives differences in receptor binding specificity between influenza B virus lineages
Source: J Virol. 2025 Nov 5;99(11):e01039-25. doi: 10.1128/jvi.01039-25 (PMC12645935; doi:10.1128/jvi.01039-25)
Supplement: Supplemental material — Figures S1 to S8; Table S1. [file jvi.01039-25-s0001.pdf]

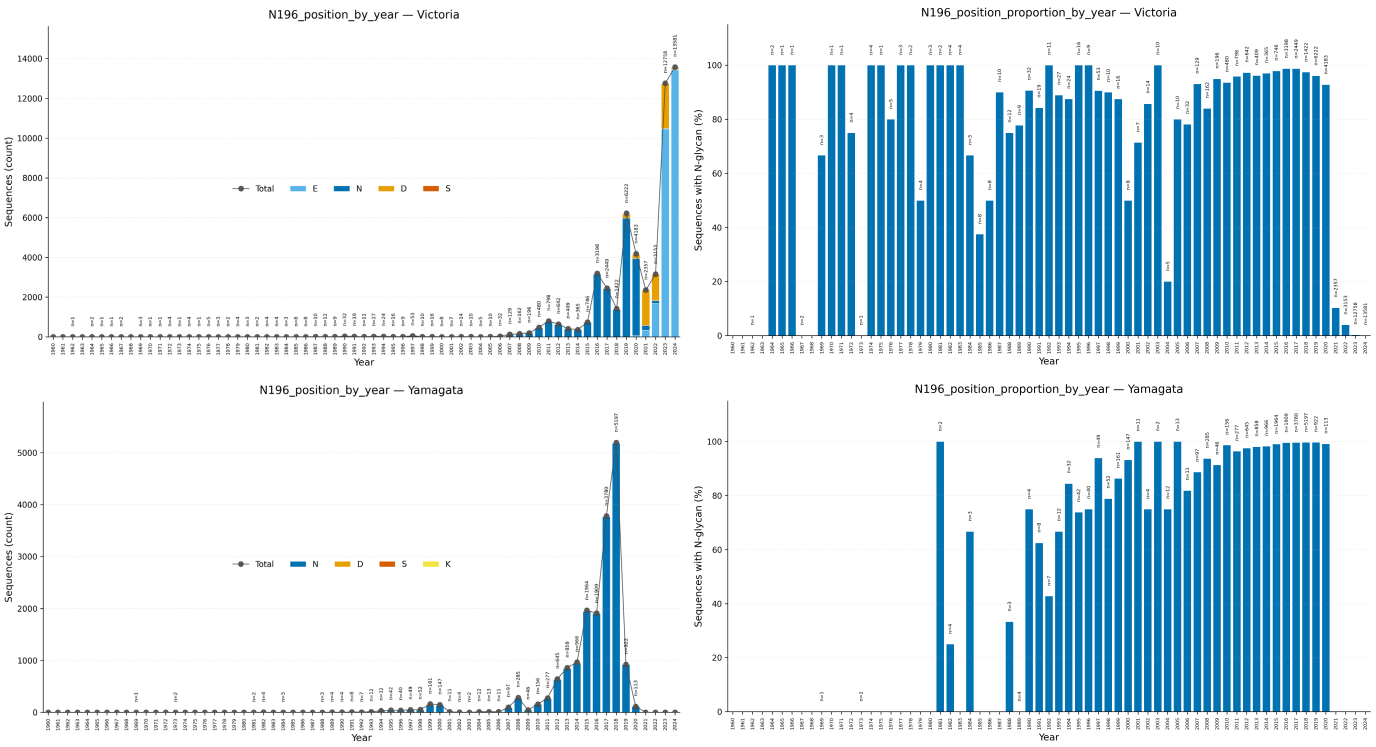

SI Figure 1. Influenza B HA temporal trends: N196 variant counts and N196 N-glycosylation prevalence by lineage (Victoria vs Yamagata, 1960–2024)

SI Table 1. Influenza B viruses used for microarray analysis. The cell lines used in this study were tMK (tertiary monkey kidney), MDCK (Madin-Darby canine kidney), and LLC-MK2 (Rhesus monkey kidney). An "x" denotes an unknown passage number, while numbers following the cell line indicate the number of passages performed in that cell line.

Number	Strain	Passage History	Lineage	EPI Accession	IRR Number	CDC #
1	B/Netherlands/1000/1962	tMK4MDCK2	Early	EPI2085776	-	-
2	B/Netherlands/1000/1966	tMK4MDCK2	Early	EPI2085779	-	-
3	B/Netherlands/1000/1969	tMK3MDCK2	Early	EPI2085780	-	-
4	B/Netherlands/1003/1974	tMK2MDCK2	Early	EPI2085785	-	-
5	B/Netherlands/1000/1977	tMK2MDCK2	Victoria	EPI2085793	-	-
6	B/Netherlands/1001/1979	tMK2MDCK2	Victoria	EPI2085803	-	-
7	B/Netherlands/223/1981	tMK1MDCK2	Victoria	EPI2085800	-	-
8	B/Netherlands/1001/1982	LLC2tMK1MDCK2	Victoria	EPI2085792	-	-
9	B/Netherlands/353/1985	tMK1MDCK2	Victoria	EPI2085806	-	-
10	B/Netherlands/1000/1989	xtMK1MDCK2	Victoria	EPI2085812	-	-
11	B/Netherlands/1000/1990	xtMK1MDCK2	Victoria	EPI2085813	-	-
12	B/Texas/43/2019	C2/C2	Victoria	-	FR-1792	-
13	B/Missouri/12/2018	MDCK2	Victoria	-	FR-1664	-
14	B/Maryland/15/2016	MDCK6	Victoria	-	FR-1576	-
15	B/Colorado/6/2017	MDCK3	Victoria	-	FR-1592	-
16	B/Hong Kong/286/2017	MDCK4	Victoria	-	FR-1619	-
17	B/Hawaii/01/2018	MDCK2	Victoria	-	FR-1661	-
18	B/Santiago/51375/2018	MDCK2	Victoria	-	-	3000683534
19	B/Washington/02/2019	MDCK2	Victoria	-	-	3026019537
20	B/Netherlands/802/1991	tMK2MDCK2	Yamagata	EPI2085817	-	-
21	B/Netherlands/25/1993	tMK1MDCK2	Yamagata	EPI2085822	-	-
22	B/Netherlands/32/1994	tMK1MDCK2	Yamagata	EPI2085824	-	-
23	B/Netherlands/8/1995	tMK1MDCK2	Yamagata	EPI2085828	-	-
24	B/Netherlands/40/1997	tMK2MDCK2	Yamagata	EPI2085833	-	-
25	B/Netherlands/3/1998	tMK2MDCK2	Yamagata	EPI2085832	-	-
26	B/Wisconsin/10/2016	MDCK2	Yamagata	-	FR-1663	-
27	B/Indiana/17/2017	MDCK2	Yamagata	-	FR-1662	-
28	B/Bolivia/111/2018	MDCK1	Yamagata	-	-	3000822961
29	B/Oklahoma/10/2018	MDCK2	Yamagata	-	FR-1660	-

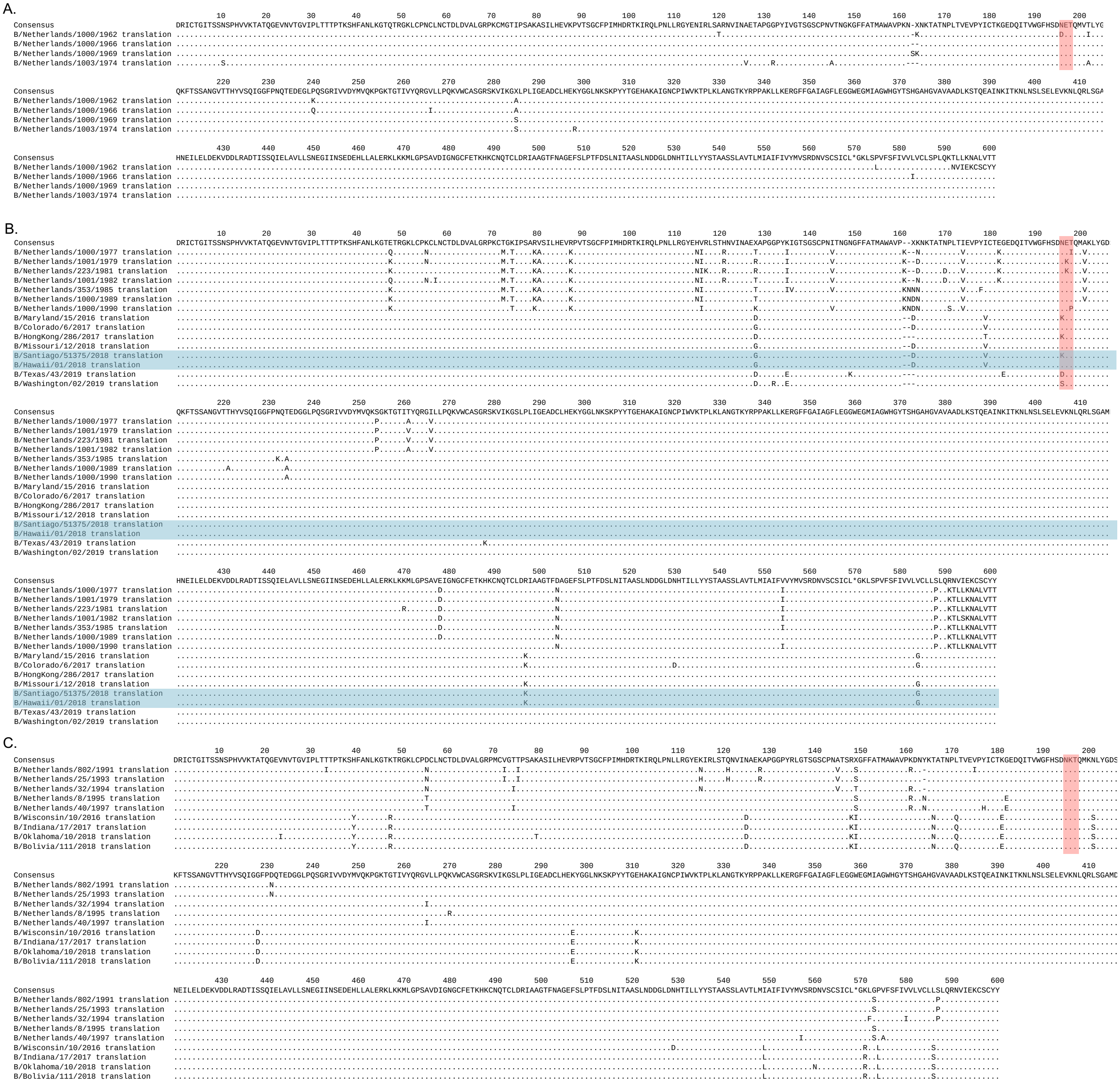

SI Figure 2. Sequence alignment of viruses. The HA of the viruses used in this study were sequenced using next-generation sequencing (NGS) and aligned in Geneious Prime. The glycosylation site of interest is highlighted in red across all alignments. The sequences of B/Santiago/51375/2018 and B/Hawaii/01/2018 are highlighted in blue to demonstrate the conservation.

A. Alignment of Early sequences.

B. Alignment of Victoria lineage sequences.

C. Alignment of Yamagata lineage sequences.

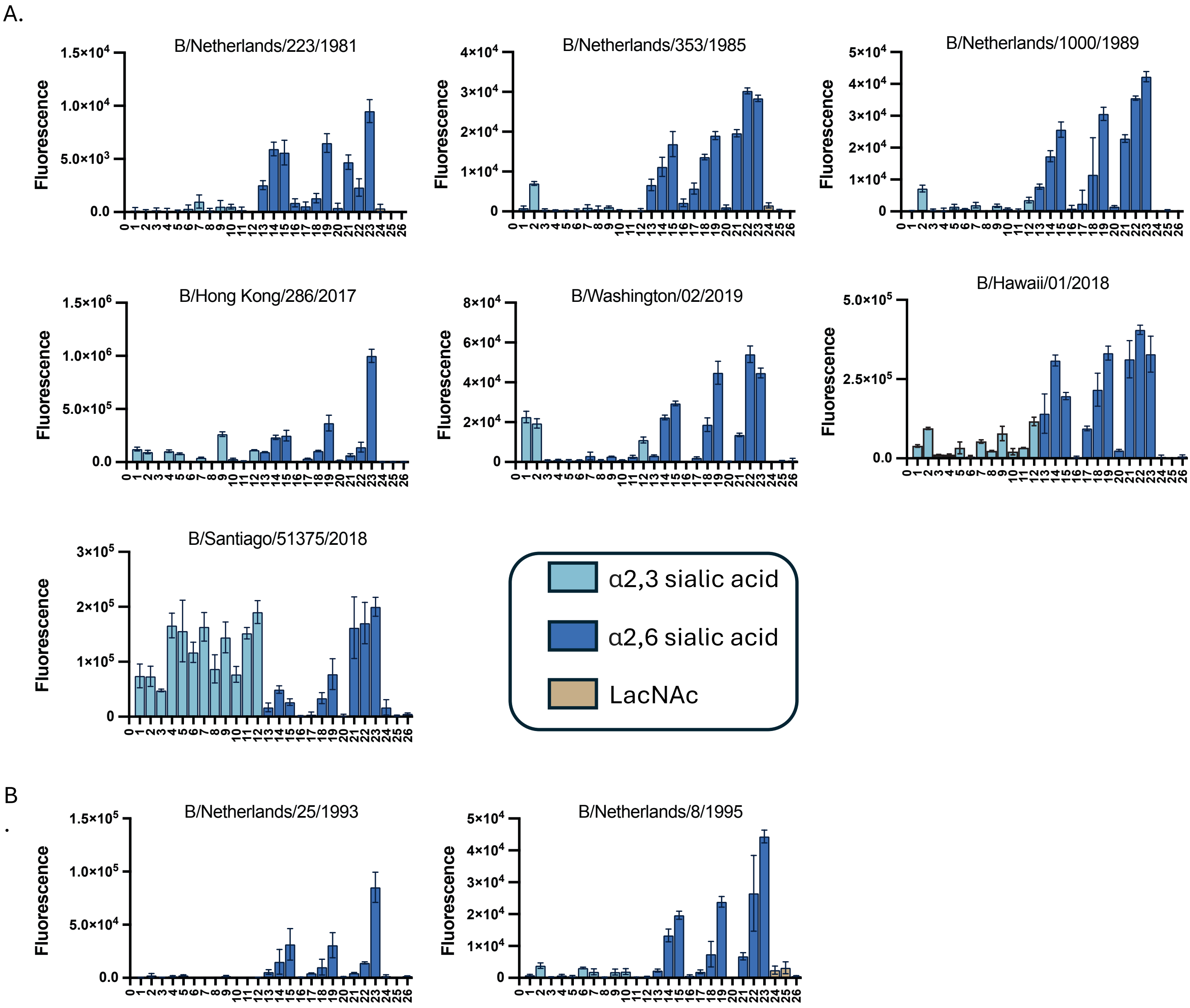

SI Figure 3. Glycan microarray analysis continued. A. Represents Victoria lineage viruses and B. Represents Yamagata lineage viruses. Bars represent the average relative fluorescence units of four replicates ± SD.

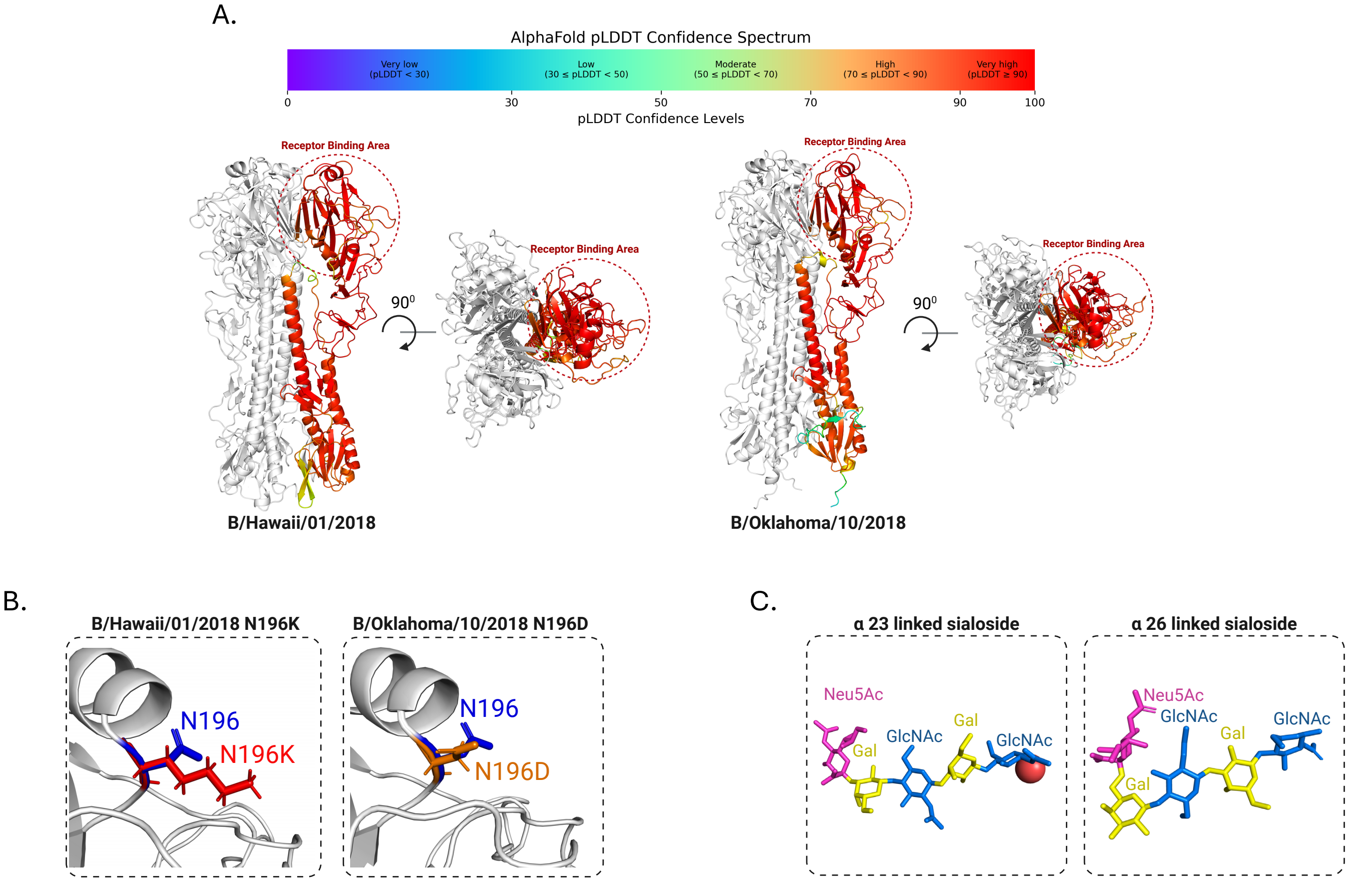

SI Figure 4. Structural modeling of variants of hemagglutinin trimer and sialic acids. A. AlphaFold pLDDT confidence spectrum mapped onto HA structures of B/Hawaii/01/2018 (Victoria) and B/Oklahoma/10/2018 (Yamagata) strains. Structural views are presented in both side and rotated orientations to show receptor-binding regions. B. Structural representation of residue 196 and its mutations (N196K and N196D, respectively). Wild-type asparagine (N196) is highlighted in blue, with mutant residues (K and D) shown in red and orange, respectively, to illustrate changes. C. Structural comparison of α2,3-linked (left) and α2,6-linked (right) sialosides, with Neu5Ac (pink), Gal (yellow), and GlcNAc (blue) color-coded.

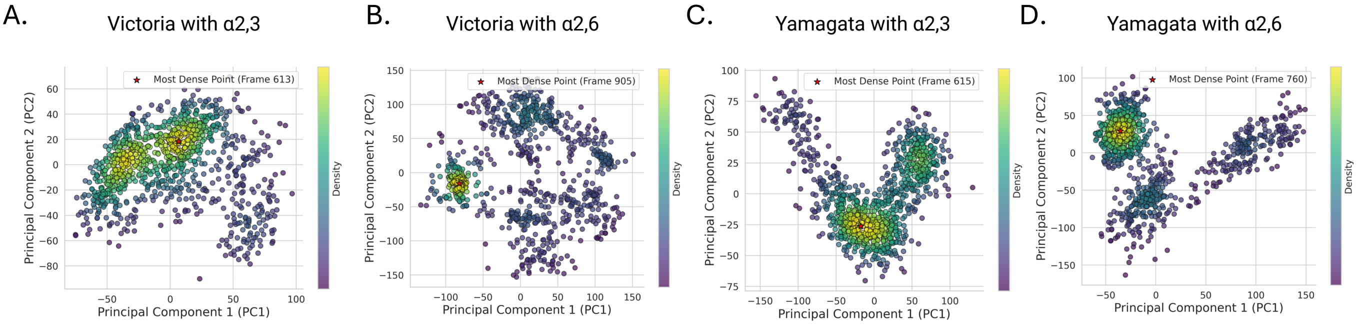

SI Figure 5. Principal Component Analysis (PCA) of Conformational Dynamics for Victoria and Yamagata Hemagglutinin with α2,3- and α2,6-Linked Sialosides. A-D. PCA plots for wild-type Victoria and Yamagata HA with α2,3 (A, C) and α2,6-linked (B, D) sialosides. Each dot represents a frame from the MD simulation, projected onto the first two principal components (PC1 and PC2), with density coloring indicating clustering of conformational states. Density values range from low (purple) to high (yellow), highlighting dominant conformational states. Most dense points (frames) are marked with a red star.

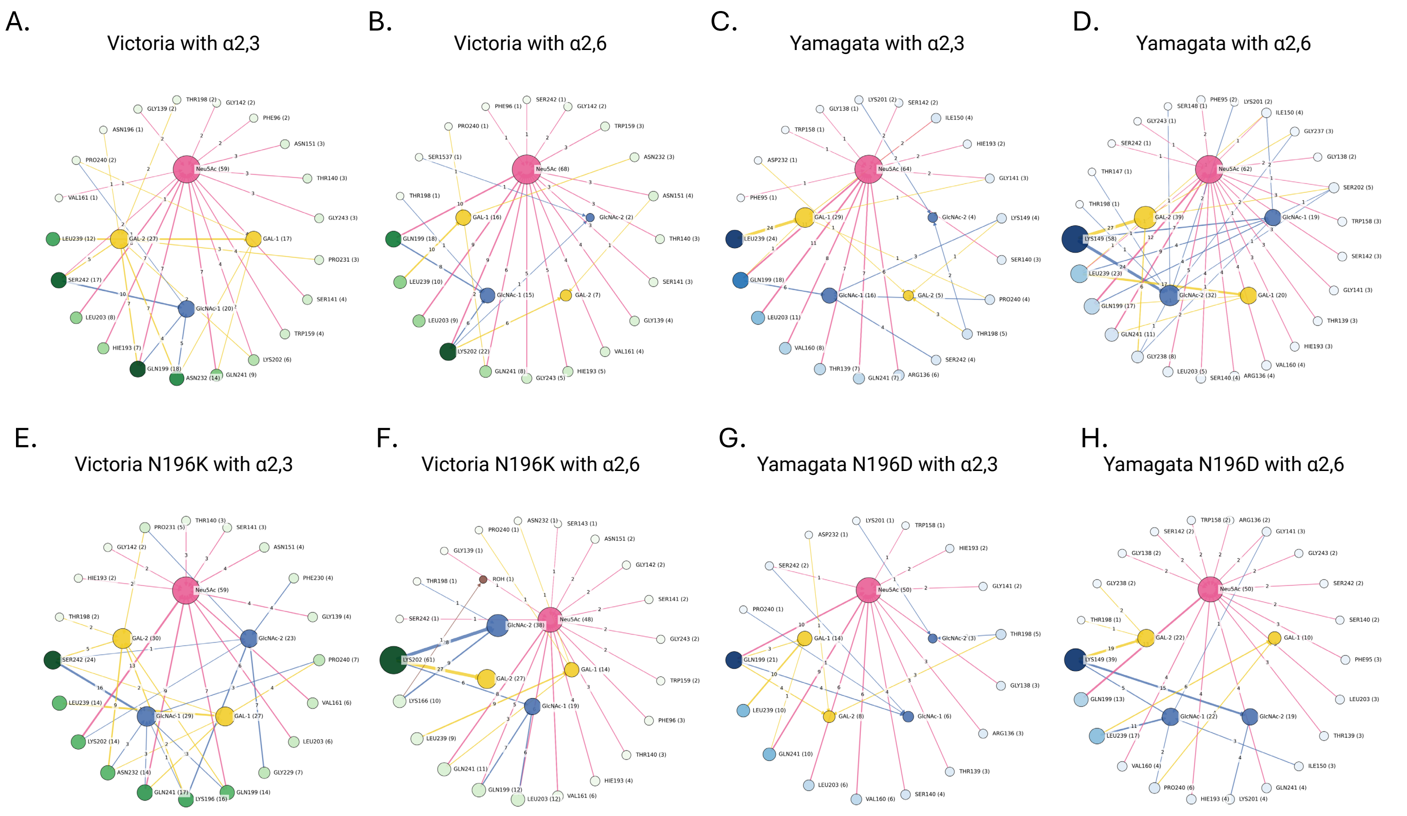

SI Figure 6. Structural interactions of HA with α2,3- and α2,6-Linked Sialic Acids. A-D. Hydrogen-bond networks for wild-type Victoria and Yamagata HA with α2,3 (blue) and α2,6-linked (green) sialosides. The thickness of the nodes corresponds to the number of interactions. E-H. Hydrogen-bond networks for mutant Victoria (N196K) and Yamagata (N196D) HA with α2,3- and α2,6-linked sialosides. Color coding of sialosides corresponds to linkage type (blue for α2,3; green for α2,6), while bonds are represented by distinct arrow styles. Residue interaction counts are noted on individual nodes.

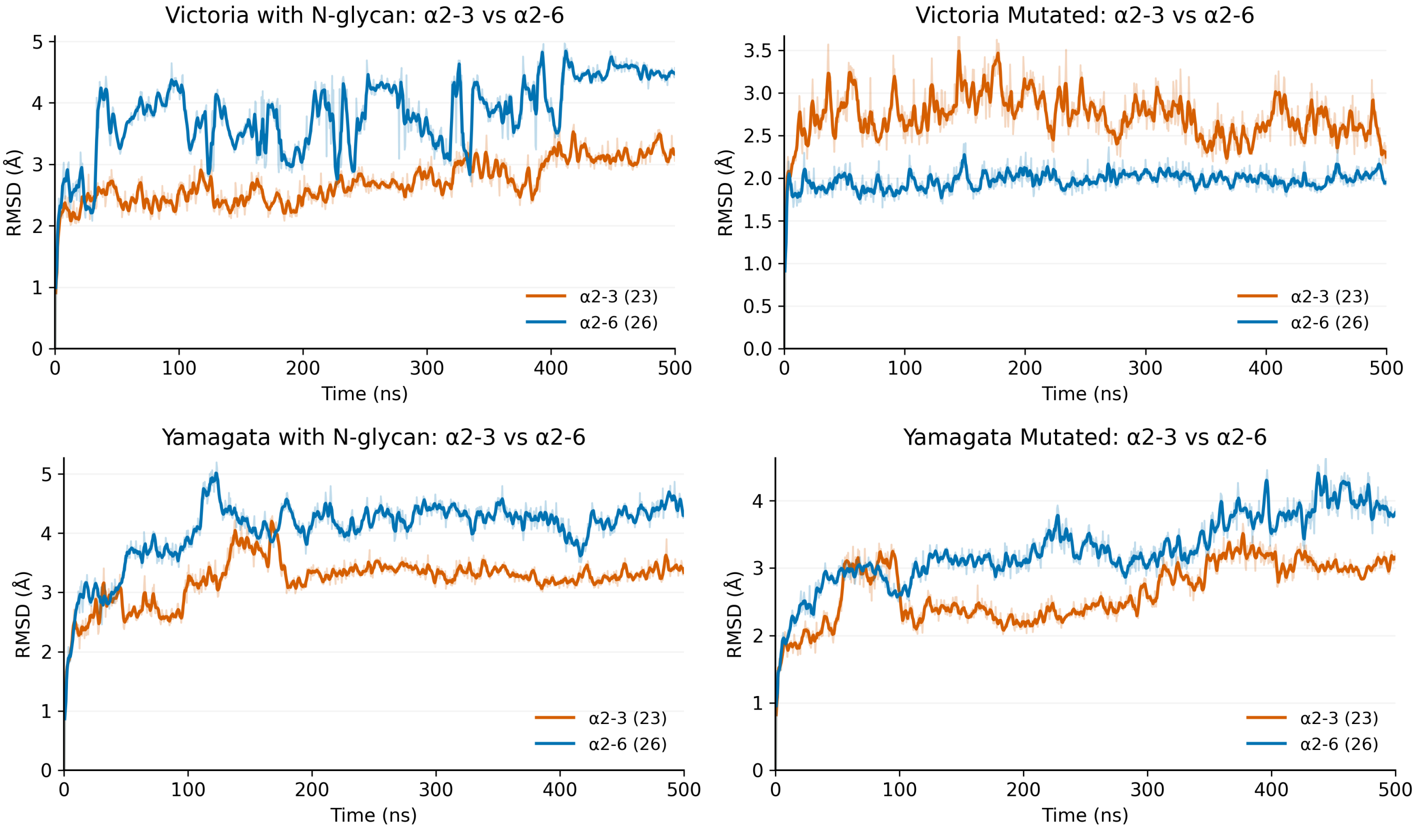

SI Figure 7. Root Mean Square Deviation (RMSD) Analysis of Victoria and Yamagata HA with α2,3- and α2,6-Linked Sialosides. Backbone RMSD (Å) over 500 ns comparing α2,3- vs α2,6-linked sialosides for Victoria (top) and Yamagata HA (bottom), shown for N-glycan–intact (left) and mutated (right) constructs. All trajectories display an initial rise followed by stable plateaus, indicating equilibration and enabling comparison of overall conformational stability.

Root Mean Square Fluctuation

A.

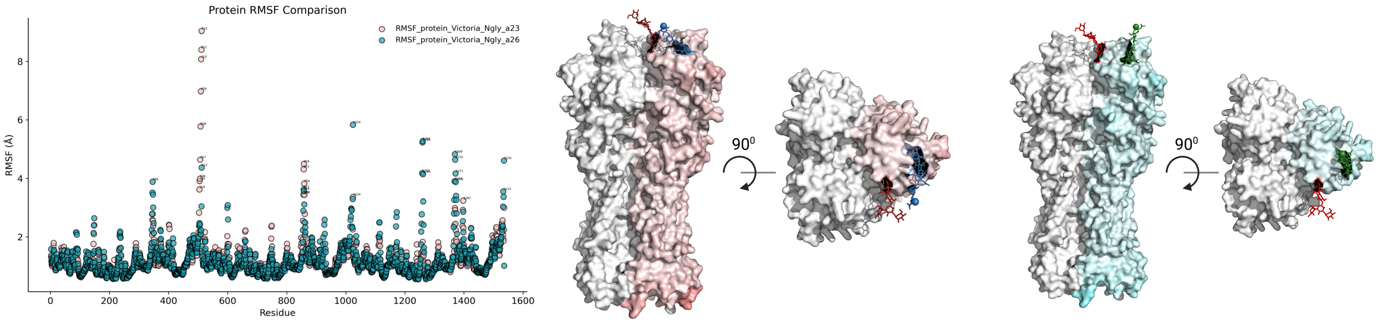

B.

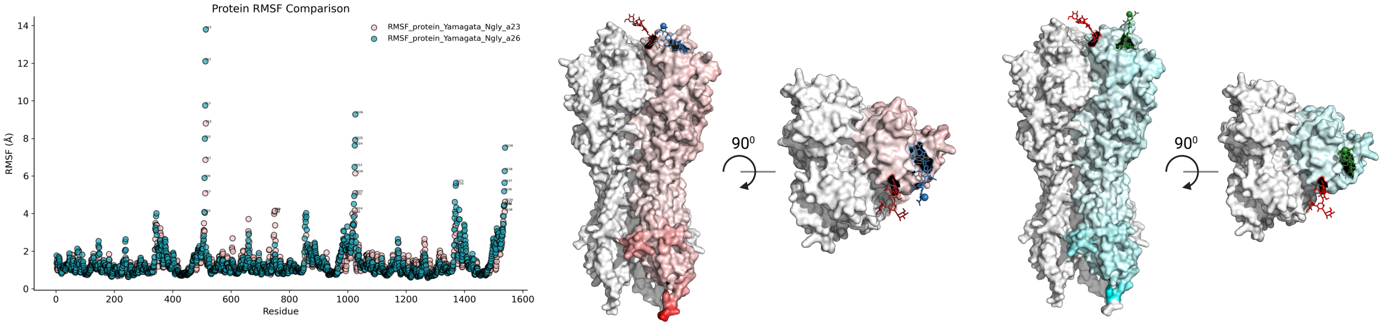

SI Figure 8. Root Mean Square Fluctuation (RMSF) Analysis of Victoria and Yamagata HA with α2,3- and α2,6-Linked Sialosides. A. RMSF profiles of Victoria HA in complex with α2,3-linked (red) and α2,6-linked (green) sialosides. The left plot shows the per-residue fluctuations across the HA backbone. On the right, RMSF values are mapped onto the HA structure, with regions of high flexibility highlighted in red. B. RMSF profiles of Yamagata HA with α2,3-linked (red) and α2,6-linked (green) sialosides. Similar to panel A, the left plot displays residue-wise fluctuations, and the right panel maps them onto the HA structure, with flexible regions highlighted.
